# Non-Coding RNA Involved in the Pathogenesis of Atherosclerosis—A Narrative Review

**DOI:** 10.3390/diagnostics14171981

**Published:** 2024-09-07

**Authors:** Kajetan Kiełbowski, Justyna Żychowska, Estera Bakinowska, Andrzej Pawlik

**Affiliations:** Department of Physiology, Pomeranian Medical University, 70-111 Szczecin, Poland; kajetan.kielbowski@onet.pl (K.K.); justynazychowskaa@gmail.com (J.Ż.); esterabakinowska@gmail.com (E.B.)

**Keywords:** atherosclerosis, cardiovascular diseases, non-coding RNA, epigenetics

## Abstract

Atherosclerosis is a highly prevalent condition associated with lipid accumulation in the intima layer of arterial blood vessels. The development of atherosclerotic plaques is associated with the incidence of major cardiovascular events, such as acute coronary syndrome or ischemic stroke. Due to the significant prevalence of atherosclerosis and its subclinical progression, it is associated with severe and potentially lethal complications. The pathogenesis of atherosclerosis is complex and not entirely known. The identification of novel non-invasive diagnostic markers and treatment methods that could suppress the progression of this condition is highly required. Non-coding RNA (ncRNA) involves several subclasses of RNA molecules. microRNA (miRNA), long non-coding RNA (lncRNA), and circular RNA (circRNA) differently regulate gene expression. Importantly, these molecules are frequently dysregulated under pathological conditions, which is associated with enhanced or suppressed expression of their target genes. In this review, we aim to discuss the involvement of ncRNA in crucial mechanisms implicated in the pathogenesis of atherosclerosis. We summarize current evidence on the potential use of these molecules as diagnostic and therapeutic targets.

## 1. Introduction

### 1.1. Atherosclerosis

Atherosclerosis describes the condition of the accumulation of fat and fibrous tissue inside the arterial wall [1]. The disorder is associated with the narrowing of the arterial lumen, which may eventually lead to organ ischemia. Furthermore, atherosclerotic lesions, which develop into plaques, can rupture, forming a thrombus that may cause acute ischemia [2]. The main factors associated with plaque rapture are thickness of the fibrous cap, lipid core size, and high macrophage density. During plaque rapture, the fibrous cap uncovers, particularly, the thrombogenic core [3,4]. The region of the cap which is the thinnest and the most exposed to macrophage infiltration holds the greatest risk of rupture [5]. Macrophages secrete many proteolytic enzymes, which are responsible for the degradation of the cap matrix built mostly with collagen fibers [6,7]. Loss of smooth muscle cells and collagen in the cap are involved in its thinning, which leads to the rupture [8]. After plaque rupture, thrombogenic factors are released from the cap, and the process of coagulation begins as activated platelets aggregate and a thrombus begins to form [9]. The clinical manifestations following plaque rapture depend on size, localization, and severity. The formation of a thrombus may lead to myocardial infarction or stroke [10]. Therefore, atherosclerosis is a background disorder of major cardiovascular diseases (CVDs). Importantly, CVDs remain the main cause of death, with an estimated 19.8 million deaths in 2022 [11,12]. Similarly, large epidemiological studies demonstrated a significant atherosclerosis prevalence and burden. According to a meta-analysis by Song et al., the prevalence of carotid plaque presence among patients 30 to 79 years old in 2020 was estimated to be 21.1% [13]. Coronary artery disease (CAD) is an atherosclerotic disease associated with inflammation. It is one of the major causes of death globally [14]. The American Heart Association estimates that 15.5 million people over 20 years old experience CAD [15]. In a study by Sata and colleagues, the authors demonstrated that the measurement of arterial stiffness and subclinical atherosclerosis parameters were associated with a 10-year absolute risk of CAD [16]. Among patients who experience transient ischemic attack or minor ischemic stroke, the presence of atherosclerosis is associated with a significantly elevated risk of major cardiovascular events in 5 years [17]. The progression and development of the disease is frequently silent until the occurrence of a major cardiovascular event or significant arterial occlusion. Due to the high frequency of patients with the silent disease [18] and the potential for serious complications, the identification of non-invasive biomarkers of early disease, as well as the introduction of novel treatment methods that would suppress atherosclerosis progression, are greatly needed.

### 1.2. Non-Coding RNA

Non-coding RNA (ncRNA) is a family of RNA molecules that are significantly implicated in the epigenetic regulation of gene expression. Epigenetic mechanisms are heritable and do not change the DNA sequence [19]. The family of ncRNA involves several subclasses, among which microRNA (miRNA), long non-coding RNA (lncRNA), and circular RNA (circRNA) are frequently investigated in the context of gene expression regulation. Over the years, researchers identified important roles of these molecules in physiology and pathophysiology. For instance, ncRNAs play a significant role in organism development [20,21]. Furthermore, they are significant elements in intercellular communication. Due to their regulatory properties in gene expression, the secretion and transport of encapsulated ncRNA to other cells change their behavior [22].

Classes of ncRNA molecules differ structurally and functionally. miRNAs are composed of approximately 22 nucleotides, and their classic mechanism of function involves binding to the 3′ untranslated region (UTR) of their target mRNA, which inhibits translation or enhances mRNA degradation. However, contrary to their classical role, evidence exists that demonstrates that these molecules can enhance gene expression [23,24]. Recent studies largely expanded knowledge about the regulation of miRNA expression. These molecules can be formed through a few pathways, but the most common and canonical one involves several RNase enzymes, such as Drosha and Dicer. Various mechanisms that regulate miRNA biogenesis were identified, such as the modification of microprocessor activity. The microprocessor, composed of Drosha and its partner DGCR8, plays a crucial role in the formation of pre-miRNA molecules. The autoregulatory function of the microprocessor, as well as the enhancement of its functionality through RNA-binding proteins, represent some of the mechanisms modulating miRNA formation [25].

The lncRNA subgroup includes molecules composed of more than 200 nucleotides. Due to their ability to bind DNA, RNA, and proteins, they are implicated in numerous regulatory mechanisms that significantly affect cellular behavior. For instance, they participate in processes associated with chromatin remodeling, which affects gene expression [26]. Furthermore, lncRNAs act as competing endogenous RNA (ceRNA) or sponges that can bind miRNA and suppress their biological functions. lncRNA molecules play a significant role in regulating the behavior of immune cells, which has implications for a large number of diseases. These mechanisms were elegantly summarized in a review by Khan et al. [27]. circRNA molecules are round-shaped molecules that can also sponge miRNAs [28]. Taking into consideration several regulatory mechanisms exerted by ncRNAs, dysregulation of their expression can significantly alter gene expression and disrupt cellular functionality.

ncRNAs are frequently investigated in the field of oncology. The altered expression of a particular molecule may enhance the expression of oncogenes or inhibit that of tumor suppressors, which drives the process of tumorigenesis [29]. However, dysregulation of ncRNAs is also observed in inflammatory diseases [30,31]. Given their dysregulated expression, ncRNA molecules can be used as diagnostic biomarkers. Moreover, as they are significant regulators of gene expression, ncRNAs are involved in the pathogenesis of diseases. Over the years, researchers have analyzed another important aspect of miRNA functionality. Single nucleotide polymorphisms (SNPs) are genetic variants that have been identified as risk factors for various conditions [32,33]. SNPs in the sequences of miRNAs or their binding genes could disrupt miRNA-mRNA interactions and, thus, alter miRNA functionality. miRNA SNPs were also found to be correlated with the presence of diseases [34,35].

Precise mechanisms participating in the pathogenesis of atherosclerosis are unknown, but several reviews have comprehensively analyzed the current literature [36]. The aim of this review is to discuss the use of ncRNAs as biomarkers in atherosclerosis and to present current evidence on the involvement of these molecules in the pathogenesis of this condition.

To perform this review, we have thoroughly searched the PubMed database. The following keywords were used: ‘atherosclerosis’, ‘non-coding RNA’, ‘microRNA’, ‘long non-coding RNA’, ‘circular RNA’, ‘extracellular vesicles’, ‘inclisiran’, ‘olpasiran’, ‘lepodisiran’, ‘SLN360’, ‘muvalapin’, and their combinations.

## 2. Non-Coding RNA and Atherosclerosis

### 2.1. MicroRNA

miRNA molecules are being studied in vitro, in vivo, and in clinical settings. The latter study design offers the opportunity to examine the diagnostic potential of these molecules. Furthermore, based on the different expressions between patients and healthy controls, together with the analysis of correlations with lipids and inflammatory mediators, these studies suggest the involvement of miRNA in pathophysiological processes associated with atherosclerosis. In vitro and in vivo studies offer a glimpse into direct mechanisms induced by miRNAs due to the ability to perform gene silencing or overexpression. However, a limited number of these investigations move into human-based studies.

In recent years, researchers began to investigate a very attractive and non-invasive liquid biopsy procedure. Using biological fluids, we could potentially diagnose a disease, gain insight into its advancement of characteristic symptoms, and monitor the progression and treatment response. ncRNAs represent molecules that could reflect the pathological conditions of the organism. In the blood, they can be encapsulated in extracellular vesicles (EVs), structures with bioactive cargo that frequently resembles the property of cells that secreted them.

**Figure 1:**
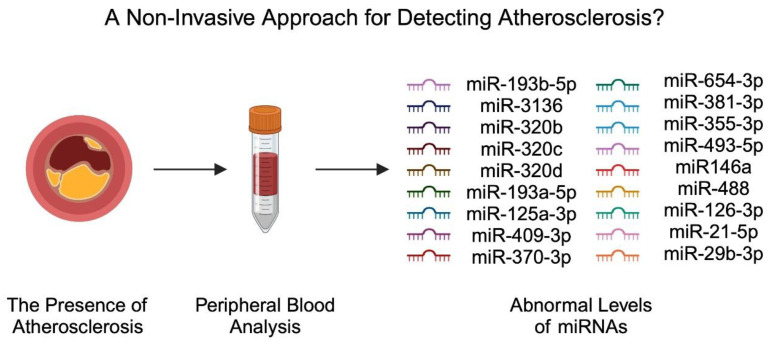
The condition of atherosclerosis is associated with altered expression of numerous miRNA molecules, which can be detected in peripheral blood. It offers a potential to implement miRNAs in the diagnostic process. Created with BioRender.com.

Recently, Brandes et al. analyzed serum EV-associated miRNAs and the plaque material of atherosclerosis patients treated surgically. Researchers detected seven upregulated EV-miRNAs in patients with CAD as compared with controls. Moreover, it was also suggested that the expression of some of the detected molecules is enhanced in carotid plaques [37]. The simultaneous expression of miRNAs in the plaques and serum may suggest that cells involved in the pathogenesis of atherosclerotic lesions secrete encapsulated miRNAs. Thus, monitoring these structures in the blood could allow for an early detection of atherosclerosis and perhaps more rapid treatment. In another study, by Hildebrandt and colleagues, the authors analyzed serum samples of 157 patients and volunteers to search for an EV-associated miRNA profile of atherosclerosis. Researchers identified different RNA molecule profiles for separate diseases caused by atherosclerosis. For instance, differentially expressed miR-215-5p, miR-199a-5p, miR-3168, miR-769-5p, and miR-582-3p were observed in patients with peripheral artery disease. CAD was associated with miR-409-3p and miR-370-3p, while a relationship between carotid artery stenosis and a group composed of miR-654-3p, miR-381-3p, miR-335-3p, and miR-493-5p was found [38]. Apart from diagnostic potential itself, monitoring miRNA levels could also suggest the severity of atherosclerosis or plaque condition. Peripheral blood concentrations of miR-146a are increased in patients with carotid atherosclerosis as compared to healthy controls. Importantly, its expression increases as the condition becomes more severe, which highlights the potential of monitoring miR-146a to evaluate stenosis progression. Its expression is also elevated in patients with more vulnerable plaques, which proved to have a predicting potential (AUC = 0.64) [39]. Other molecules with elevated expression in patients with atherosclerosis involve miR-488, miR-27a, miR-133a, and miR-203, among others [40,41]. miR-126-3p, miR-21-5p, miR-29b-3p, and miR-223-3p represent some of the molecules downregulated in patients with CAD, which also showed potential diagnostic values [42] (Figure 1). Atherosclerosis is frequently clinically silent until the arterial lesions achieve certain dimensions. Moreover, the condition represents a common comorbidity in a number of diseases, such as autoimmune disorders [43]. Recently, monitoring miRNA has been suggested as a marker of subclinical atherosclerosis in Sjogren’s disease. Zehrfeld et al. showed a positive correlation between miR-92a-3p levels and carotid intima–media thickness [44].

The development of atherosclerosis is strongly associated with lipid disturbances. Specifically, a higher blood concentration of non-HDL lipoproteins is considered a major pathophysiology factor associated with the progression of the disease. Low-density lipoproteins (LDLs) and very low-density lipoproteins (VLDLs) can migrate into the arterial intima, where they undergo modifications, such as oxidation. Modified lipoproteins then enhance foam cell formation and endothelial dysfunction, leading to the initiation and progression of atherosclerotic plaques [45]. Recent studies have addressed how interventions targeting dyslipidemia and atherosclerosis affect miRNA expression. Successes in this field would allow for the introduction of response biomarkers. Monitoring the expression or concentrations of these molecules could perhaps lead to a more rapid drug change, which could eventually result in more personalized treatment methods and better outcomes. For instance, treatment with a high dose of rosuvastatin is associated with an improved lipid profile, together with the lower expression of miR-33b-5p in atherosclerotic plaques obtained through endarterectomy. This observation follows an expected mechanism of action, as miR-33b-5p is one of the molecules that downregulate the expression of ABCA1 [46]. Analyses of lipids and circulating miRNAs might lead to the identification of response markers, which has been examined in a study by Mangas and colleagues. The authors showed that a panel comprising miR-376c-3p, miR-376a-3p, let-7c-5p, let-7d-5p, and let-7f-5p could be used to detect statin-intolerant patients (AUC 0.936) [47].

The identification of mechanisms and molecules that stimulate dyslipidemia is crucial to understanding the pathophysiology of atherosclerosis. In vitro and in vivo studies were performed to search for potential associations between miRNAs and pathways regulating lipid metabolism. One such molecule is the proprotein convertase subtilisin kexin 9 (PCSK9), which is a mediator of cholesterol metabolism. Specifically, it stimulates the degradation of LDL receptor (LDLR), which is present in hepatocytes. As a result, circulating LDLs are less effectively eliminated, and their levels are increased [48]. Targeting PCSK9 with monoclonal antibodies like evolocumab and alirocumab is a known method to lower LDL concentrations [49]. As miRNAs regulate gene expression by binding to their target mRNAs, they could potentially downregulate the expression of PCSK9 and induce similar effects to those observed in PCSK9 inhibitors.

Firstly, Naeli et al. demonstrated that miR-191, miR-222, and miR-224 can bind the 3′UTR region of PCSK9. In HepG2 cells, overexpression of these miRNAs reduced that of PCSK9 [50]. Using lipid nanoparticles, the stimulation of Ldlr^+/−^ mice with miR-224 was associated with a 15% decrease in circulating LDL [51]. Frequently, miRNAs regulate the expression of a large number of target genes, being involved in a broad interaction network. Importantly, this indicates that they mediate the activity of several pathways. As a result, a single molecule can promote both beneficial and detrimental effects, depending on the cellular context. Regarding miR-222, Bazan and colleagues suggested that this miRNA could protect from plaque rupture. Among patients who underwent urgent carotid endarterectomy, the expression of miR-222 in plaque shoulder was significantly reduced [52]. However, in a different study, miR-222-5p could stimulate vascular smooth muscle cell (VSMC) dysfunction [53]. miR-483-5p is another molecule that targets PCSK9. Its overexpression in HepG2 cells stimulated the uptake of LDL molecules. In humans, the expression of miR-483-5p was negatively correlated with cholesterol serum levels [54].

**Figure 2:**
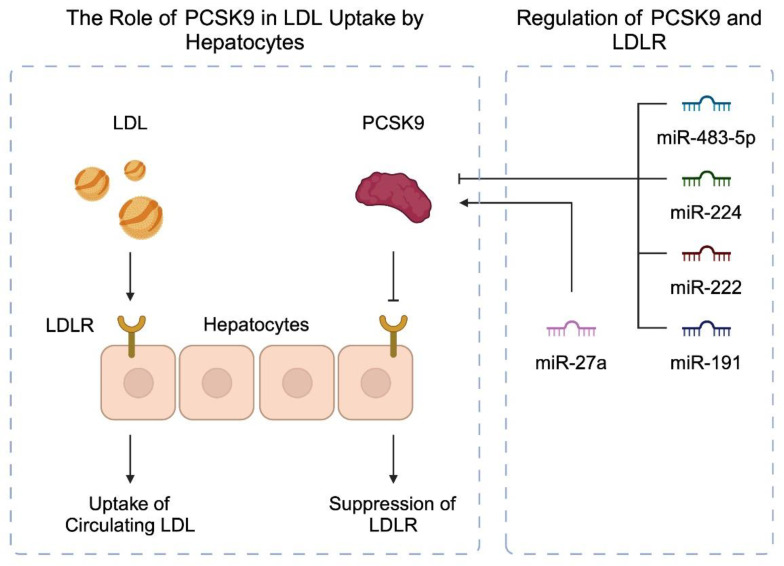
miRNAs regulate the expression of molecules involved in lipid metabolism, thus affecting the progression of atherosclerosis. This figure schematically demonstrates the miRNA-mediated regulation of PCSK9. Created with BioRender.com.

By contrast to the molecules described above, other miRNAs can enhance the expression of PCSK9, thus stimulating LDLR degradation and the progression of atherosclerosis. miR-27a was found to target molecules involved in LDLR endocytosis, including LDLR-related protein 6 (LRP6) and LDLR-adapter protein 1 (LDLRAP1). Moreover, the molecule increased the expression of PCSK9 [55]. Intriguingly, the use of PCSK9 immunogenic peptide in mice was associated with a significant decrease in miR-27a expression [56]. LDLR plays a very important role in the metabolism of LDL from plasma. It has been shown that genetic dysfunctions of LDLR are associated with increased cardiovascular risk through an increase in plasma LDL concentration [57]. Recently, a new LDLR regulator, the (pro)renin receptor [(P)RR], was identified. Interestingly, Wang et al. proved that miR-148a strongly affects (P)RR, reducing its expression, which also reduces the concentration of LDLR in Huh7 and HepG2 cells and ultimately leads to a reduction in cellular LDL uptake [58]. Additionally, there are studies that indicate that obese mice have higher concentrations of miR-148a in the adipose tissue and liver [59,60]. Furthermore, miR-152 is also associated with the regulation of (P)RR expression [61]. Another interesting finding is that LDLR abundance can be modulated by miR-33a-3p. Additionally, the expression of ANGPTL3, an LPL inhibitor, is directly inhibited by miR-33a-3p, contributing to the reduction in LDL in plasma [62,63]. Factors that regulate LDLR may also be miR-224 or miR-520d. Overexpression of these miRs leads to a decrease in LDLR protein and reduced LDL binding [51]. Other microRNAs involved in LDLR expression include miR-128-1, miR-185, and miR-27a/b [64]. Figure 2 summarizes the involvement of miRNAs in PCSK9 and LDL metabolism.

Another molecule highly implicated in lipid metabolism is lipoprotein lipase (LPL). It is considered to induce both pro- and anti-atherogenic effects, depending on the presence and cellular origin. LPL present in the arterial wall takes part in the hydrolysis of triglycerides (TGs) in lipoproteins. Consequently, the production of free fatty acids (FFAs), together with cholesterol-rich remnant lipoproteins, contributes to atherosclerosis progression [65]. Similarly to PCSK9, miRNAs also regulate the expression of LPL. Firstly, administration of miR-590 in apoE^−/−^ mice was associated with reduced atherosclerotic plaque lesions. Additionally, in these animal models, miR-590 reduced plasma cholesterol and decreased lipid accumulation in peritoneal macrophages. Mechanistically, the miRNA molecule downregulated macrophage LPL expression [66]. By contrast, several molecules stimulate the expression of LPL, thus enhancing lipid accumulation, pro-inflammatory conditions, and the development of atherosclerosis. Stimulation of LPL activity seems to be an indirect mechanism induced by miRNAs. One of the molecules linking miRNAs and LPL is angiopoietin-like 4 (ANGPTL4). Decreased plasma levels of ANGPTL4 in patients suffering from angina and undergoing coronary angiography were associated with more advanced coronary stenosis [67]. Injection of ANGPTL4 into ApoE^−/−^ mice fed with a high-fat diet suppressed the progression of atherosclerosis [68]. Importantly, the protein suppresses the activity of the LPL through the unfolding of its hydrolase domain [69]. Lan and collaborators found that ANGPTL4 was targeted and downregulated by miR-134. Simultaneously, it was associated with enhanced activity of LPL. Therefore, miR-134 potentially enhances plaque formation and atherosclerosis-associated inflammation through ANGPTL4 [70]. In another study, performed by Cheng and colleagues, the authors demonstrated that miR-182 has pro-atherogenic potential and regulates the activity of LPL. Mechanistically, it targets histone deacetylase 9 (HDAC9), a negative regulator of LPL. Similarly to the miR-134/ANGPTL4 axis, the miR-182/HDAC9 pathway enhanced plaque formation in ApoE^−/−^ mice [71]. Interaction between miR-467b and hepatic LPL was observed in the context of hepatic steatosis. Downregulation of miR-467b was observed in hepatic tissues of mice fed with high-fat diets, and was associated with insulin resistance [72]. Hepatic steatosis is a metabolic condition indirectly associated with atherosclerosis [73,74].

The development of atherosclerosis is highly correlated with the processes of lipid accumulation. Scavenger receptors, such as CD36, allow for the uptake of lipoproteins. Specifically, CD36 present on macrophages can bind to oxidized LDL, which subsequently is internalized and contributes to foam cell formation [75]. Studies demonstrated that miRNA molecules regulate the expression of scavenger receptors, thus influencing lipid accumulation. In a large analysis performed by Rachmawati and collaborators, the authors analyzed miRNA databases, and identified tens and hundreds of molecules targeting CD36 [76]. The precise involvement of miRNA in CD36-mediated foam cell formation was examined in other studies as well. In THP-1-derived macrophages, miR-758-5p was found to target the 3′UTR region of CD36 and mediate ox-LDL uptake [77]. Like previously discussed pathways, miRNAs act indirectly and mediate the expression of CD36 as well. Peng et al. showed that miR-133a reduces macrophage lipid uptake by targeting testicular orphan nuclear receptor 4 (TR4), a nuclear receptor that enhances the activity of CD36-dependent foam cell formation [78].

**Figure 3:**
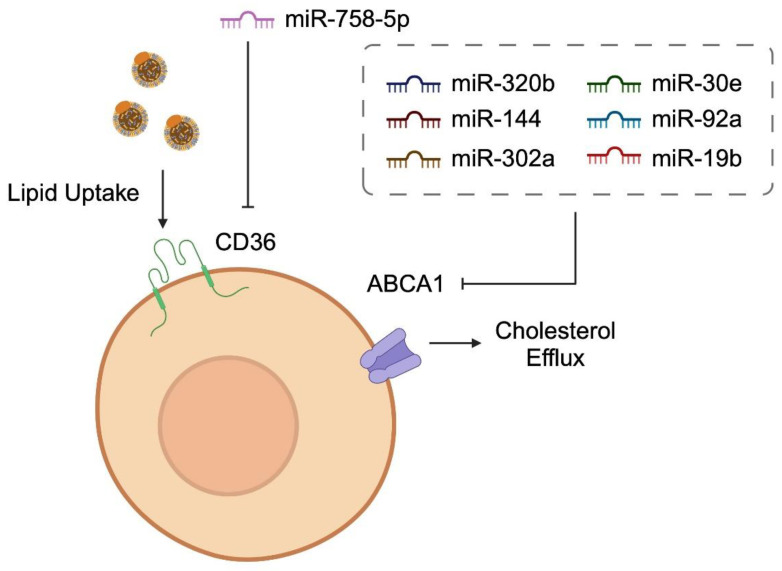
A schematic illustration demonstrating the influence of selected miRNA molecules on lipid uptake and cholesterol efflux in macrophages. Created with BioRender.com.

Another method that is associated with cholesterol transport and atherosclerosis is cholesterol efflux. This process is mediated by the ATP-binding cassette (ABC) transporters such as ABCA1. These transporters enhance the secretion of HDL, and the cholesterol efflux capacity has been associated with lower cardiovascular risk [79,80]. Modulating the expression of ABC transporters is another mechanism that could be targeted in the treatment of atherosclerosis. The expression of ABCA1 is also mediated by miRNAs. miR-320b was found to target ABCA1/G1 transported in macrophages. Accordingly, its overexpression suppressed cholesterol efflux. Intriguingly, the expression of miR-320b was upregulated in peripheral blood mononuclear cells (PBMCs) obtained from patients with CAD [81]. Additionally, 16-week treatment with anti-miR-144 of Ldlr^−/−^ mice fed with a Western diet increased the protein expression of ABCA1. Simultaneously, the upregulation of ABCA1 was accompanied by a 20% increase in HDL cholesterol. This treatment was associated with reduced plaque formation, as compared to animals in different cohorts [82]. Other miRNAs regulating the expression of ABCA1 include miR-30e, miR-92a [83], miR-19b [84], miR-33a [85], and miR-302a [86], among others. Figure 3 schematically presents the involvement of miRNAs in lipid uptake and cholesterol efflux. Furthermore, Table 1 summarizes the role of miRNAs in regulating molecules associated with lipid involvement and transportation.

As previously mentioned, ncRNA can be encapsulated in extracellular vesicles (EVs), such as exosomes, to mediate paracrine signaling. Over the years, researchers have shown the important role of EVs and ncRNAs associated with EVs in the pathogenesis of various diseases [87,88]. Accumulating studies are being published that examine the involvement of exosomal miRNA in the pathogenesis of atherosclerosis. To begin with, the activity of ncRNA-based cargo frequently depends on the type of secreting cells. Macrophages represent a good example, as these cells are typically classified as the pro-inflammatory M1 and anti-inflammatory M2 phenotypes. In atherosclerosis, the M1 macrophage variants are considered enhancers of the disease. Therefore, treatment strategies that can suppress M1 polarization and enhance that of M2 are considered beneficial in atherosclerosis [89,90]. As the biological effects of exosomes carrying miRNAs seem to resemble the source cells, vesicles secreted from the M1 macrophages are found to drive the progression of this arterial condition. Li and colleagues showed that EVs obtained from M1 macrophages could promote the progression of atherosclerosis by increasing lipid and inflammation markers in mice. These findings were suggested to occur, at least partly, through the activity of miR-185-3p, which targets Smad7 [91]. As encapsulated miRNAs take part in intercellular signaling, macrophages accumulate EVs and are affected by these molecules as well. Under pro-inflammatory conditions, endothelial cells secrete exosomes that affect macrophage behavior. Specifically, these structures enhance M1 macrophage polarization and lipid accumulation. miRNA present in the EVs could mediate these observations. One hundred four differentially expressed miRNAs have been observed when comparing endothelial cell exosomes derived under normal and pro-inflammatory conditions [92].

By contrast, exosomes derived from naïve or anti-inflammatory macrophages induce different effects. EVs obtained from naïve bone marrow-derived macrophages significantly reduce the extent of plaque necrotic core in ApoE^−/−^ mice. Moreover, treatment with exosomes obtained from macrophages stimulated with IL-4, which enhances the M2 phenotype, reduces areas infiltrated by macrophages and simultaneously increases the M2 markers in residual cells. Treatment with IL-4 alters miRNA cargo of exosomes and increases the presence of anti-inflammatory molecules [93]. Interestingly, miRNAs associated with EVs take part in the pathogenesis of atherosclerosis in other models as well. Recently, endothelial cell-derived exosomal miR-126 and miR-212 were suggested to be involved in the activation of monocytes in the irradiation model of atherosclerosis [94]. Thus, these lines of evidence demonstrate a crucial role of exosome-mediated communication between endothelial cells and macrophages. However, EV-associated miRNAs secreted by other cells are also involved in the pathophysiology of atherosclerosis. For instance, steatotic hepatocytes secrete EVs containing miR-1, which stimulate inflammatory responses in endothelial cells and are involved in the pathogenesis of atherosclerosis [95]. ncRNAs could be used as diagnostic molecules. A recently published study by Blaser and colleagues further confirms this hypothesis. Using disease-specific proteomics, the authors analyzed carotid endarterectomy specimens and demonstrated 80 differently enriched extracellular vesicle-associated miRNAs between artery atherosclerosis and valve stenosis [96].

### 2.2. Long Non-Coding RNA

Similarly to mRNA, lncRNA is transcribed by polymerase II, but its expression is lower in tissues [97]. It is composed of over 200 nucleotides and is linear, which distinguishes it from other ncRNAs [98]. The lncRNA class includes overlapping sense lncRNAs, antisense RNAs, and intergenic noncoding RNAs (lincRNAs) [99]. Unusual lncRNA structures influence gene expression through various mechanisms, including regulating transcription and translation, acting as sponges for miRNAs, controlling interactions between proteins, regulating signaling pathways, and modulating chromatin through histone modification, among others [100]. Recent reports draw attention to the association of lncRNAs in the development of atherosclerosis. As previously mentioned, atherosclerosis occurs as a result of the disruption of many biological processes, such as inflammation, apoptosis, angiogenesis, adipogenesis, and arterial endothelial function [101].

An lncRNA called ANRIL is located on chromosome 9p21 and is an antisense gene for cyclin-dependent kinase inhibitor 2B (CDKN2B). Recent studies suggested that ANRIL could be a component of the TNF-α/NF-κB pathway, which is strongly associated with inflammatory responses of cells implicated in atherosclerosis progression. An increase in ANRIL expression leads to endothelial dysfunction through the TNF-α-NF-κB-ANRIL/YY1-IL6/8 axis. TNF-α stimulates the activity of NF-κB, which upregulates ANRIL. Importantly, ANRIL interacts with YY-1, which is an important transcription factor involved in inflammatory processes by increasing the expression of interleukins and COX2 genes [102]. Additionally, ANRIL increases the expression of several molecules involved in the metabolism of glycolipids, potentially leading to the development of atherosclerosis [103]. Moreover, it has been suggested that the ANRIL rs4977574 gene polymorphism may influence the occurrence of atherosclerosis. The rs4977574 intron locus genotype is involved in the regulation of the production of circular and linear ANRIL. Circular ANRIL promotes the activation of the p53 protein, which leads to an increase in apoptosis, while linear ANRIL inhibits the apoptosis process by repressing CDKN2A and CDKN2B, which are tumor suppressors. It has been proven that the rs4977574-GG genotype increases the risk of atherosclerosis, which may be related to the predominance of linear ANRIL [104,105]. ANRIL inhibits let-7b, which leads to increased human umbilical vein endothelial cell (HUVEC) proliferation and angiogenesis. Mechanistically, ANRIL regulates the let-7b/TGF-βR1 signaling pathway. Reducing the expression of TGF-βR1 may lead to the formation of neointima, which mediates the formation of atherosclerotic plaques [106].

**Figure 4:**
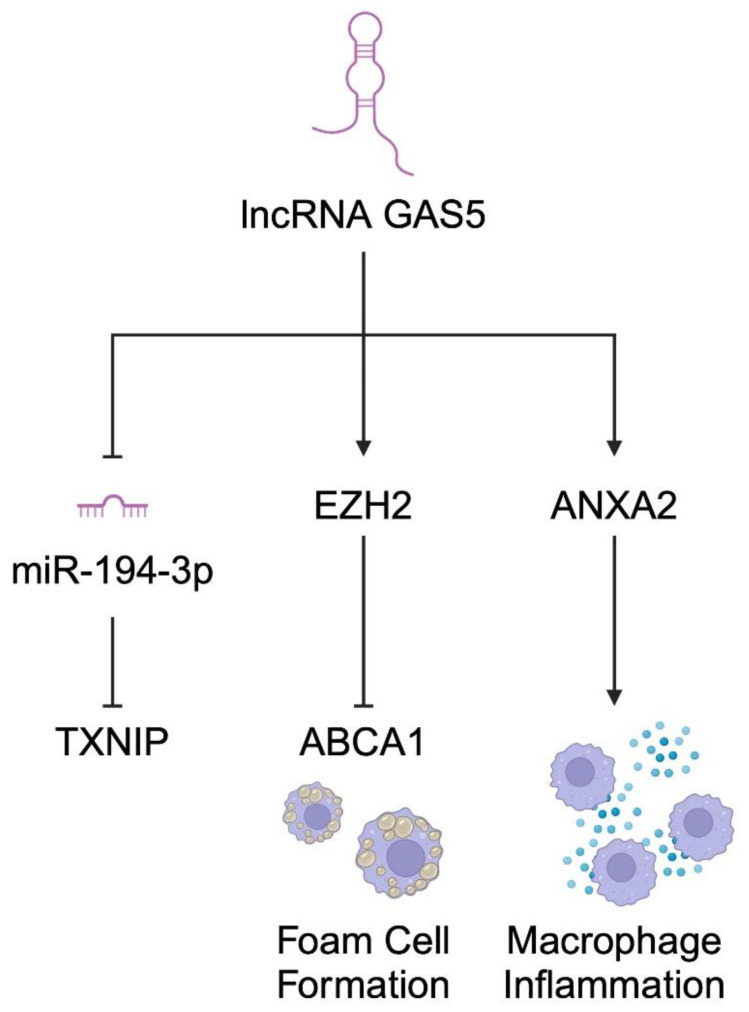
A schematic illustration demonstrating several mechanisms implicated by lncRNA GAS5 in the pathogenesis of atherosclerosis. Created with BioRender.com.

lncRNA growth arrest-specific 5 (GAS5) is another molecule that is suggested to be involved in the progression of atherosclerosis [107]. For instance, it stimulates lipid accumulation in macrophages, thus contributing to the formation of foam cells [108]. lncRNA GAS5 was found to be implicated in several mechanisms associated with atherosclerosis. Li et al. described how GAS5 can induce atherosclerosis through the GAS5/miR-194-3p/TXNIP pathway. The authors showed that the expression of GAS5 was elevated in rats with atherosclerosis. The molecule regulated the expression of TXNIP by acting as a sponge for miR-194-3p [109]. Interestingly, it has been proven that ANXA2, a calcium-regulated protein that binds phospholipids and belongs to the annexin X family, is a target for GAS5 in macrophages. ANXA2 deficiency in mice inhibited the development of atherosclerosis and endothelial cell proliferation. It regulates several mechanisms, including the transduction of inflammatory cell signaling, thus mediating the proliferation of macrophages into plaques. Moreover, ANXA2 increases the release of TNF-α, IL-1β, and IL-6 by binding to the TLR4 receptor located on macrophages [110,111,112]. In another study, Meng et al. described that GAS5 binds to the enhancer of zeste homolog 2 (EZH2), which is a histone methyltransferase. Mechanistically, GAS5 enhanced EZH2 activity, which negatively regulated ABCA1 expression. Consequently, the axis enhanced intracellular lipid accumulation [108]. Shen et al. pointed out that GAS5 can act as a sponge of miR-135a, which affects the molecule Janus kinase 2 and toll-like receptor 4 (TLR4). Consequently, a lower expression of miR-135a indicates a higher risk of plaque formation by disturbing lipid metabolism in macrophages [113]. Additionally, GAS5 has been shown to inhibit the expression of miR-21, which enhanced that of PDCD4 proteins. PDCD4 proteins promote the development of atherosclerosis by increasing the apoptosis of HUVEC. Increased PDCD4 expression was also found in macrophage-derived foam cells [114]. Therefore, due to the potential involvement in the pathogenesis of atherosclerosis, GAS5 could be a promising target in the diagnosis and therapy of this arterial condition (Figure 4).

As is widely known, large amounts of amino acids in the diet increase the risk of atherosclerotic plaque formation. Qu et al. proved that increased amino acid supply in mice activates mTORC1 signaling in macrophages. This leads to the disruption of processes such as autophagy and lipid biosynthesis. lncRNA Gpr137b-ps disrupts the interactions between G3BP and HSC70, the presence of which has been proven in macrophages. Disruption of the interaction of G3BP with HSC70 leads to the activation of mTORC1 signaling, which disturbs macrophage autophagy. Disturbed autophagy leads to the formation of necrotic cores and contributes to the formation of atherosclerotic plaques by modulating the inflammatory response. Interestingly, increased autophagy leads to the destruction of atherosclerotic plaques by breaking down lipids in foam cells [115].

INKILN, another lncRNA, has potential pro-inflammatory effects in VSMCs. Mechanistically, INKILN stabilizes the MKL1 protein, which affects the p65/NF-κB pathway and leads to the activation of VSMC inflammation. Consequently, this leads to arterial disease, which may destabilize the atherosclerotic plaque [116]. Interestingly, the lncRNA PELATON may influence the pathogenesis of atherosclerosis by increasing the expression of CD36 on macrophage cells, thereby increasing the uptake of dead cells and lipoproteins and the production of reactive oxygen species [117].

### 2.3. Circular RNA

Unlike lncRNA, the structure of circRNA is composed of a closed-loop strand. Thus, it is an ncRNA with much greater stability due to the lack of a polyadenylated tail and 5′-3′ polarity. The much greater resistance of circRNAs to ribonucleases than lncRNAs may indicate the potential function of these ncRNAs as disease biomarkers. circRNAs perform various functions that influence gene expression. They act as sponges for miRNAs, reducing the level of miRNA-mRNA interactions, and can also act as sponges and scaffolds for proteins, e.g., the HuR protein [118].

Triska et al. conducted a review of 140 studies from 2016–2022 and showed that as many as 76.8% of 95 isolated circRNAs are overregulated in patients with atherosclerosis and 79% of them have pro-atherogenic potential. Interestingly, circHIPK3 has been found to be both downregulated and upregulated in patients with atherosclerosis. Intriguingly, only 10 of the 140 studies did not show that circRNA acted as a sponge for miRNA [119]. circRNA-0044073 has become a potential ncRNA that may be associated with the occurrence of atherosclerosis. It broadly affects the expression and activity of miR-107, JAK1, p-STAT3, c-myc, and Bcl-2. Mechanistically, circRNA-0044073 can act as a sponge of miR-107, which affects the molecule JAK1 and p-STAT3. Consistently, higher JAK1 and p-STAT3 expression is associated with an increase in IL-8 in atherosclerotic cells and with greater vascular cell adhesion. Additionally, an increase in c-myc and Bcl-2 contributes to the deregulation of apoptosis in atherosclerotic plaques [120]. circ_102541 regulates the expression miR-296-5p, which targets PLK1. In atherosclerosis, an increase in circ_102541 expression was demonstrated, which reduced that of miR-296-5p, stimulating PLK1. Interestingly, transfection of sh-circRNA_102541 caused the opposite effect, reducing PLK1 in HUVEC cells and increasing the efficiency of apoptosis [121]. Increased PLK1 expression is associated with enhanced proliferation of VSMCs in the inner membrane of blood vessels [122]. Due to its proliferation-inducing effects, the activity of PLK1 is associated with tumorigenesis as well. Luo et al. showed that treatment of HUVECs with ox-LDL increased levels of circRNA-PTPRA. Conversely, the knockdown of this circRNA abolished the pathological effects of modified lipoprotein, such as an increase in inflammation or a decrease in cell viability, by regulating miR-671-5p [123].

Atherosclerosis may co-occur with asthma. IgE and mast cells cooperate in this process. In a recent report by Yang et al., the authors concluded that through stimulation of exosomal circRNA CDR1as secreted by mast cells, IgE influences endothelial dysfunction by dysregulating adhesion molecules intercellular adhesion molecule-1 (ICAM-1) and vascular cell adhesion molecule-1 (VCAM-1) [124,125]. These findings indicate that although interleukins may be helpful in the treatment of severe asthma, they do not necessarily have a beneficial effect on the comorbid atherosclerosis in these patients. This sheds new light on the pathogenesis and potential treatment of both diseases [125].

As we mentioned earlier, linear ANRIL is associated with the development of atherosclerosis. However, what is interesting is that circRNAs can be created as a result of the so-called back-splicing of linear transcripts. This indicates both differences and similarities in the action of these two ncRNAs. However, the theory that lncRNAs influence the pathogenesis of atherosclerosis and circRNAs inhibit this process is confirmed. Ribosome biogenesis in VSMCs is regulated by circRNAs. circRNA ANRIL binds to the lysine-rich domain of pescadillo zebrafish homologue 1 (PES1), thereby reducing rRNA maturation, leading to p53 activation and apoptosis, and protecting against atherosclerosis [126]. In summary, both lncRNAs and circRNAs have promising diagnostic and therapeutic potential; however, further research on these molecules is needed (Table 2).

## 3. Potential Clinical Implications

In this paper, we have discussed the involvement of ncRNA in the pathogenesis of atherosclerosis. Current evidence suggests that the altered expression of these molecules is involved in the pathophysiology of this arterial condition. Therefore, the development of agents targeting ncRNA is expected to induce beneficial changes in affected cells.

Importantly, several RNA-based drugs have been developed for the treatment of dyslipidemia and other conditions. Firstly, apart from miRNA, small interfering RNAs (siRNAs) are another group of molecules involved in the mechanisms known as RNA interference (RNAi). siRNA molecules depend on a 100% complementarity, and siRNA-based therapeutics such as patisiran and givosiran represent a breakthrough in drug development [127]. Inclisiran is an siRNA-based therapeutic targeting PCSK9 approved in the EU for the treatment of primary hypercholesterolemia. In hepatocytes, inclisiran binds to the PCSK9 mRNA and suppresses translation [128]. The efficacy and safety of inclisiran were evaluated in the ORION clinical trials. In a recently published analysis of the ORION-11 trial, which evaluated the use of siRNA-based drugs in patients without prior cardiovascular events, inclisiran significantly reduced LDL-C levels. Regarding the safety analysis, more patients in the study group experienced adverse events (AEs; 92.9% vs. 83.8%) and serious AEs (20.4% vs. 12.4%) [129]. Furthermore, a pooled analysis of ORION-10 and ORION-11 trials also proved the efficacy of inclisiran in reducing atherogenic lipoproteins in patients after myocardial infarction [130]. In in vivo experiments, the drug was directly demonstrated to reduce atherosclerotic plaque formation [131]. Intriguingly, inclisiran was found to be superior in combination with statins in suppressing LDL-C concentrations, as compared to the cohort receiving the usual care [132]. Olpasiran is another RNA therapeutic examined in the context of atherosclerosis. Its mechanism of action involves the inhibition of the expression of the apolipoprotein (a) gene, which in turn disrupts the formation of lipoprotein(a) in the liver. According to the recently published results of the OCEAN[a]-DOSE clinical trial by O’Donoghue et al., researchers analyzed the use of olpasiran in patients with a history of atherosclerotic CVD. The authors observed a significant decrease in lipoprotein(a) concentrations in the study group. Furthermore, reduced levels of LDL-C and apolipoprotein-B were noted. Importantly, the AE rates were found to be similar between the cohorts [133]. Lepodisiran (LY3819469) is another siRNA-based therapeutic that reduces the levels of lipoprotein(a). A clinical trial that included patients with elevated levels of lipoprotein(a) and without cardiovascular events revealed that treatment with lepodisiran dose-dependently substantially reduced lipoprotein(a) concentrations. As it was a phase 1 trial, whose primary outcome was the analysis of safety, researchers observed that the treatment was well tolerated [134]. Similar results were observed in other phase 1 studies examining siRNA therapeutics, SLN360 and muvalaplin [135,136]. Thus, current evidence shows promising results regarding the use of siRNA-based therapeutics. More phase 2 and 3 clinical trials are greatly needed to further analyze the efficacy and safety of these therapies on larger cohorts of patients. Perhaps, these agents could be used in primary or secondary cardiovascular prevention. Interestingly, miRNA-based therapeutics are also being designed and examined. For instance, the use of an miR-34a mimic was evaluated in cancer settings [137], while miravirsen, an oligonucleotide targeting miR-122, was evaluated in hepatitis C virus infection [138]. If this new generation of RNA-based drugs will prove their efficacy and safety, a new era of individualized treatment might emerge.

## 4. Conclusions and Future Perspectives

To conclude, ncRNAs are involved in a broad number of interactions with molecules regulating lipid metabolism and transportation, inflammatory mediators, as well as other members of the other ncRNA classes. Current evidence suggests that members of the ncRNA family contribute to lipid metabolism by regulating the expression of PCSK9, LPL, scavenger receptors, and ABCA1 transporters. Consequently, the abnormal expression of ncRNAs is associated with lipid levels and foam cell formation. Moreover, due to their immunoregulatory properties, they also mediate inflammatory responses in cells involved in the pathogenesis of atherosclerosis.

Over the years, accumulating evidence has been collected on the involvement of ncRNA in the pathophysiology of atherosclerosis. However, the precise responses induced by these molecules remain unknown. Future studies should try to identify the complex network of interactions, as ncRNA frequently affects various signaling pathways. Published studies demonstrated that ncRNAs are dysregulated in animal models and patients with atherosclerosis. Due to the subclinical character of early processes of plaque formation, the use of ncRNAs as biomarkers of early atherosclerosis might eventually prevent major cardiovascular events. Furthermore, studies should examine if monitoring RNA expression could help in identifying treatment responses to drugs used in patients with dyslipidemia. Moreover, understanding the ncRNA-dependent regulatory mechanisms involving lipid accumulation might result in the implementation of novel treatment methods in the future. Several siRNA-based therapeutics are being currently examined in patients with dyslipidemia. Future clinical trials should further investigate their efficacy and safety in combination with other lipid-lowering agents. Perhaps, more miRNA-, lncRNA-, or circRNA-based therapeutics might be developed that will show benefits in patients with atherosclerosis.

## Figures and Tables

**Table 1:** A summary of the roles of selected microRNAs in mediating the progression of atherosclerosis.

miRNA Molecule	Study Design	Target Associated with Atherosclerosis	Potential Mechanisms Associated with Atherosclerosis Regulation	References
miR-191	In vitro	PCSK9	Targeting PCSK9 in hepatocytes can improve the expression of LDLR.	[50]
miR-222	In vitro	PCSK9	Targeting PCSK9 in hepatocytes can improve the expression of LDLR.	[50]
miR-224	In vitro In vivo	PCSK9	Targeting PCSK9 in hepatocytes can improve the expression of LDLR. Application of miR-224 to Ldlr^+/−^ mice was associated with a 15% decrease in circulating LDL.	[50,51]
miR-483-5p	In vitro	PCSK9	Overexpression of miR-483-5p enhanced LDL uptake.	[54]
miR-27a	In vivo	LRP6, LDLRAP1	miR-27a disrupts LDLR endocytosis by targeting molecules involved in this process. Moreover, it increases the expression of PCSK9.	[55]
miR-590	In vivo	LPL	Through downregulating macrophage LPL expression, miR-590 could reduce atherosclerotic plaque formation.	[66]
miR-134	In vivo	AGPTL4	By targeting ANGPTL4, miR-134 stimulates the activity of LPL and enhances plaque progression.	[70]
miR-182	In vivo	HDAC9	miR-182 enhances the activity of LPL and atherosclerosis progression by targeting HDAC9, which negatively regulates LPL.	[71]
miR-467b	In vivo	LPL	miR-467b was found to target LPL in hepatocytes, and downregulation of this molecule was associated with hepatic steatosis and insulin resistance.	[72]
miR-758-5p	In vitro	CD36	miR-758-5p was found to mediate cholesterol accumulation by THP-1-derived macrophages.	[77]
miR-133a	In vitro	TR4	By targeting nuclear receptor TR4, miR-133a indirectly suppresses lipid uptake mediated by CD36.	[78]
miR-320b	In vitro In vivo	ABCA1/G1	miR-320b targeted ABC transporters and reduced cholesterol efflux from macrophages.	[81,82]
miR-144	In vivo	ABCA1	Treatment of Ldlr^−/−^ mice fed with a Western diet with miR-144 inhibitor increased ABCA1 expression and HDL cholesterol levels and decreased atherosclerotic plaque lesions.	[82]

**Table 2:** A summary of lncRNAs and circRNAs and their mechanisms implicated in the pathogenesis of atherosclerosis.

lncRNA	Study Design	Association with Atherosclerosis Pathophysiology	Mechanism of Action/Pathway	References
GAS5	In vitro In vivo	- endothelial damage - promoting inflammation - regulation of intracellular lipid accumulation - disturbance of lipid metabolism in macrophages - increasing the apoptosis of HUVEC	- GAS5/miR-194-3p/TXNIP - GAS5-ANXA2 - GAS5-EZH2-ABCA1 - GAS5/miR-135a - GAS5/miR-21/PDCD4	[108,109,110,113,114]
ANRIL	In vitro	- endothelial damage - disturbance of glycolipid metabolism - neointimal formation	- TNF-α-NF-κB-ANRIL/YY1-IL6/8 - VAMP3, ET-1, ADIPOR1, C11ORF10 - let-7b/TGF-βR1	[102,103,106]
Gpr137b-ps	In vivo	- disturbance of macrophage autophagy	- G3BP/HSC70/mTORC1	[115]
INKILN	In vitro	- pro-inflammatory effects in VSMC	- MKL1/p65/NF-κB	[116]
circRNA	Study Design	Target	Mechanism Associated with Atherosclerosis	References
circRNA-0044073	In vitro	miR-107	Decrease levels of miR-107 via sponging and activation of JAK/STAT pathway	[120]
circ_102541	In vitro	miR-296-5p	Regulates miR-296-5p expression, which targets PLK1	[120]
circRNA-PTPRA	In vitro	miR-671-5p	Decrease circRNA-PTPRA expression; decrease cell viability and inflammation via miR-671-5p regulation	[123]

## Data Availability

Not applicable.
